# Outpatient and inpatient anticoagulation therapy and the risk for hospital admission and death among COVID-19 patients

**DOI:** 10.1016/j.eclinm.2021.101139

**Published:** 2021-09-24

**Authors:** Sameh M. Hozayen, Diana Zychowski, Sydney Benson, Pamela L. Lutsey, Jasmin Haslbauer, Alexandar Tzankov, Zachary Kaltenborn, Michael Usher, Surbhi Shah, Christopher J. Tignanelli, Ryan T. Demmer

**Affiliations:** a Department of Medicine, Division of General Internal Medicine, Assistant Professor of Medicine, Hospitalist, University of Minnesota, Mayo Building, 420 Delaware Street, SE, 6 Floor, Room D694, Minneapolis, MN 55455, United States; b Department of Medical Education, University of Minnesota, United States; c Division of Biostatistics, School of Public Health, University of Minnesota, Minneapolis, MN, United States; d Division of Epidemiology and Community Health, School of Public Health, University of Minnesota, Minneapolis, MN, United States; e Pathology, Institute of Medical Genetics and Pathology, University Hospital Basel, University of Basel, Switzerland; f Department of Hematology and oncology, Mayo Clinic, Arizona, United States; g Department of Surgery, University of Minnesota, Minneapolis, MN, United States; h Institute for Health Informatics, University of Minnesota, Minneapolis, MN, United States; i Department of Surgery, North Memorial Health Hospital, Robbinsdale, MN, United States; j Department of Epidemiology, Mailman School of Public Health, Columbia University, New York, NY, United States

**Keywords:** COVID-19, Anticoagulation, Hospitalization, Mortality, D-dimer, Inpatient, Outpatient, COVID-19, coronavirus disease 2019, OPAC, outpatient persistent anticoagulation therapy, IPAC, inpatient anticoagulation therapy, SARS-CoV-2, severe Acute Respiratory Syndrome Coronavirus-2, rt-PCR, reverse transcriptase-polymerase chain reaction, EHR, electronic health records, EMR, electronic medical records, DOAC, direct oral anticoagulant, mg/dl, milligram per deciliter, ACEi, angiotensin-converting enzyme inhibitors, ARBs, angiotensin receptor blockers, MI, prior myocardial infarction, VTE, venous thromboembolism, IRB, institutional review board, DIC, disseminated intravascular coagulation, HIT, heparin-induced thrombocytopenia, HR, hazard ratio, SD, standard deviations, SE, standard errors, CI, confidence intervals, %, percentage, (n), number, SBP-min, minimum systolic blood pressure, SpO_2_-min, minimum oxygen saturation, T1DM, type 1 diabetes mellitus, T2DM, type 2 diabetes mellitus, COPD, chronic obstructive pulmonary disease, CKD, chronic kidney disease, CO2, carbon dioxide, HCT, hematocrit, RDW, red blood cell distribution width, SBP, systolic blood pressure, WBC, white blood cell

## Abstract

**Background:**

Coronavirus disease 2019 (COVID-19) is associated with a hypercoagulable state. Limited data exist informing the relationship between anticoagulation therapy and risk for COVID-19 related hospitalization and mortality.

**Methods:**

We evaluated all patients over the age of 18 diagnosed with COVID-19 in a prospective cohort study from March 4th to August 27th, 2020 among 12 hospitals and 60 clinics of M Health Fairview system (USA). We investigated the relationship between (1) 90-day anticoagulation therapy among outpatients before COVID-19 diagnosis and the risk for hospitalization and mortality and (2) Inpatient anticoagulation therapy and mortality risk.

**Findings:**

Of 6195 patients, 598 were immediately hospitalized and 5597 were treated as outpatients. The overall case-fatality rate was 2•8% (*n* = 175 deaths). Among the patients who were hospitalized, the inpatient mortality was 13%. Among the 5597 COVID-19 patients initially treated as outpatients, 160 (2.9%) were on anticoagulation and 331 were eventually hospitalized (5.9%). In a multivariable analysis, outpatient anticoagulation use was associated with a 43% reduction in risk for hospital admission, HR (95% CI = 0.57, 0.38–0.86), *p* = 0.007, but was not associated with mortality, HR (95% CI=0.88, 0.50 - 1.52), *p =* 0.64. Inpatients who were not on anticoagulation (before or after hospitalization) had an increased risk for mortality, HR (95% CI = 2.26, 1.17–4.37), *p =* 0.015.

**Interpretation:**

Outpatients with COVID-19 who were on outpatient anticoagulation at the time of diagnosis experienced a 43% reduced risk of hospitalization. Failure to initiate anticoagulation upon hospitalization or maintaining outpatient anticoagulation in hospitalized COVID-19 patients was associated with increased mortality risk.

**Funding:**

No funding was obtained for this study.

**Research in context**

**Evidence before this study**

We searched the MEDLINE database of references and abstracts using the words: anticoagulation, COVID-19, inpatient, outpatient, outcomes, mortality between February 2020 through March 2021. We didn't limit the search to publications in the English language. We found a meta-analysis of five studies showing that anticoagulation was not associated with mortality in hospitalized COVID-19 patients. We also found a study showing that anticoagulation was associated with reduced mortality in elderly outpatients diagnosed with COVID-19.

**Added value of this study**

Outpatients with COVID-19 who were on outpatient anticoagulation at the time of diagnosis experienced a 43% reduced risk of hospitalization. Failure to initiate anticoagulation upon hospitalization or maintaining outpatient anticoagulation in hospitalized COVID-19 patients has been associated with increased mortality risk.

**Implications of all the available evidence**

Randomized controlled trials for anticoagulation therapy among both inpatients and outpatients are urgently needed to address the type, dosage, and duration of anticoagulation for COVID-19 patients.

Alt-text: Unlabelled box

## 1 Introduction

Coronavirus disease 2019 (COVID-19) caused by Severe Acute Respiratory Syndrome Coronavirus-2 (SARS-CoV-2) affects multiple cell types with systemic effects outside the respiratory tract [1], [2], [3]. Observational studies have noted adverse thromboembolic events in those with COVID-19, prompting further research on the prevention and treatment of thrombosis [[4], [5], [6], [7], [8]].

The pathophysiology of hypercoagulability in COVID-19 is incompletely understood. However, the increased risk of thromboembolic events is likely related to traditional risk factors and mechanisms unique to COVID-19, such as increased inflammation, hypoxia, and endothelium inflammation [9,10]. The binding of SARS-CoV-2 to the target host cell generates the release of inflammatory cytokines, promoting immune cell migration to the site of tissue damage [11]. These activated immune cells exacerbate endothelial damage through increased vascular leak and micro thrombus formation [12,13]. The higher mortality rates observed among COVID-19 patients with elevated D-dimers may be related to these mechanisms [7,14,15].

Anticoagulants are indicated in patients with venous thromboembolism and atrial fibrillation with the shortest duration of three months for provoked venous thromboembolism [16,17]. We decided to study the impact of persistent outpatient anticoagulation (OPAC) on the risk of adverse outcomes in the setting of COVID-19. A retrospective Italian study involving seventy elderly patients found that outpatient anticoagulation was associated with reduced COVID-19 mortality [18]. In an uncontrolled study from the USA, one hundred patients on outpatient anticoagulation had lower thrombotic complications and less severe disease when they are diagnosed with COVID-19 [19].

While some studies have explored the role of outpatient anticoagulation, most have focused on inpatient anticoagulation strategies to reduce thrombotic events and mortality. For example, in a study of 4389 hospitalized COVID-19 patients from the USA, anticoagulation lowered in-hospital mortality and intubation, although there was no statistically significant difference in patients’ outcomes with prophylactic versus therapeutic anticoagulation strategies [20]. In another retrospective study of 395 COVID-19 inpatients in New York City requiring mechanical ventilation, in-hospital mortality was 29% for those treated with therapeutic anticoagulant versus 62% for those who did not receive any anticoagulation [21]. In a Chinese single-center study of hospitalized COVID-19 patients, 99 of 449 were treated with prophylactic dose low molecular weight heparin. Heparin treatment was not associated with mortality overall; however, heparin treatment was associated with a lower risk of death among patients with elevated D-dimer or an elevated sepsis-induced coagulopathy score [22]. Few studies have examined the impact of risk-stratified initiation of anticoagulation based on illness severity using age, gender, comorbid conditions, vital signs, and D-dimer value among patients hospitalized with COVID-19 despite their widespread use [[23], [24], [25], [26], [27], [28]]. To that effect, the improved outcomes of hospitalized COVID-19 patients on anticoagulation negated prior concerns in the scientific community for disseminated intravascular coagulopathy (DIC) that were reported earlier in the pandemic [29]. This paper defined inpatient anticoagulation (IPAC) as the initiation of anticoagulation for prophylactic, escalated prophylactic or therapeutic dose, or continuation of outpatient anticoagulation.

To further explore the impact of anticoagulation on COVID-19 outcomes, we leveraged data from a large sample of adult COVID-19 patients from a single hospital system in the upper Midwest. Based on the improved outcomes among hospitalized patients exposed to anticoagulation, we hypothesized that outpatient anticoagulation (OPAC) would be associated with decreased risk of hospital admission and mortality among patients on anticoagulation before COVID-19 diagnosis compared to patients who were not. We also hypothesized that IPAC would decrease the risk of mortality among inpatients compared to patients who are not on any anticoagulation.

## 2 Methods

### 2.1 Design and source population

**Fig. 1:**
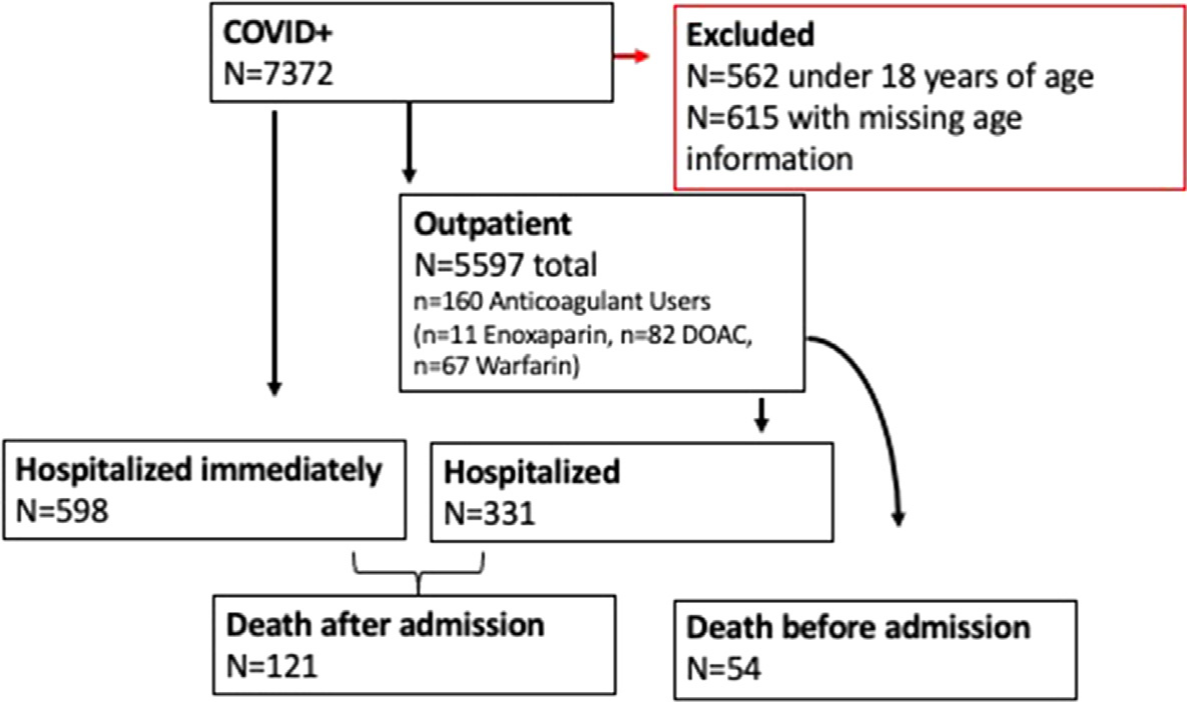
Flow chart of patient enrollment and progression to hospitalization and death.

We conducted a cohort study among patients primarily managed in a large academic health care system of 12 Midwest hospitals and 60 primary care clinics between March 4th and August 27th, 2020. Inclusion criteria included being actively managed as of March 4th, 2020, age ≥ 18 years, and nasopharyngeal reverse transcriptase-polymerase chain reaction (rt-PCR) confirmed COVID-19 infection. This resulted in a total sample of *n =* 6195 patients for analysis and included 5597 individuals initially treated as outpatients (Fig. 1). To account for patient transfers across hospitals or clinics, data were pooled across different electronic health records (EHRs), and a unique patient identifier was created accounting for the clinic, emergency department, or hospital. In cases where a patient had been encountered in two different EHRs, the most comprehensive EHR record was utilized. The COVID-19 subject-oriented database includes individual-level data for patients with rt-PCR-confirmed COVID-19, covering a diverse range of ages, races, ethnicities, and geographic regions within the Midwest as described in prior publications from this group [30,31]. All patients that opted out of research were excluded from the analysis. This study was approved by the University of Minnesota institutional review board (IRB) (STUDY00001489), which provided a waiver of consent for this study. The IRB has issued the waiver of the consent process for this study since our study fulfilled the following four criteria: i. involved no more than minimal risk to the subjects; ii. The waiver will not affect the subjects' rights adversely; iii. We could not carry out the study without the waiver; iv. Finally, we could not carry out the study without using biological and epidemiological information in an identifiable format.

### 2.2 Medication assessment

Medications were abstracted from electronic medical records (EMR). The use of outpatient angiotensin-converting enzyme inhibitors/angiotensin receptor blockers (ACEi/ARBs) was also explored due to the proposed impact on COVID-19 outcomes. Other antihypertensive medications were not individually examined. Among patients with positive rt-PCR initially treated as outpatients, those prescribed any anticoagulant class including warfarin, a direct oral anticoagulant (DOAC, i.e., apixaban, rivaroxaban, dabigatran, edoxaban), or enoxaparin in the immediate 90 days prior to COVID-19 diagnosis were included. We chose 90 days since this is the shortest duration for persistent outpatient anticoagulation (for provoked venous thromboembolism) [16]. Among inpatients, we created an integrated definition of outpatient/inpatient anticoagulant therapy as follows: (i) patients who never used anticoagulation, neither inpatient or outpatient use; (ii) patients who were initiated on anticoagulation upon admission; (iii) patients who were on anticoagulation as an outpatient and were continued as an inpatient. Based on our institutional policy, universal anticoagulation was required for all COVID-19 patients admitted to the hospital starting May 2020 unless there was a contraindication. We stratified the patients deemed high risk based on the D-dimer (> 10 times the upper limit of normal), admission to intensive care units or being on high flow oxygen, active cancer, and prior history of venous thromboembolism. Those who qualified to be high risk received escalated prophylactic dose anticoagulation with 0.5 mg/kg of enoxaparin or low-intensity heparin infusion as permitted by their renal clearance. Patients who did not qualify as high risk received routine weight-based enoxaparin or heparin as determined by their glomerular filtration rate. Those who were on therapeutic anticoagulation prior to admission continued at the same intensity. Anticoagulation was held if platelet count was below 30,000 or if there was another contraindication such as active gastrointestinal bleeding or recent intracerebral hemorrhage. Patients would be prescribed anticoagulation with a DOAC for 14 days after discharge. Interruption of anticoagulation was planned prior to invasive procedures and also for a very limited number of bleeding events. Physicians’ adherence to our institutional anticoagulation algorithm was more than 90%. Prior to the date of institutional policy enforcement (May 2020), anticoagulation was used based on the discretion of the treating physician, given concerns for DIC in the scientific community as detailed above [29]. Our rate of adherence for venous thromboembolism prophylaxis in COVID-19 patients hospitalized prior to that date was similar to the national average of about 50% [32].

### 2.3 Outcome assessment

Our analysis was focused on two groups. Our first group (outpatient COVID-19) consisted of patients diagnosed with COVID-19 who were deemed to be stable enough to be managed as outpatients. For this group, our primary outcome was hospital admission for all causes and in-hospital and out-of-hospital death on or before August 27th, 2020. We compared patients who received OPAC to patients who did not. Our second group (inpatient COVID-19) consisted of all COVID-19 patients admitted to the hospital for all causes regardless of whether they were immediately admitted to the hospital or initially managed as an outpatient. For this group, our primary outcome was death. We compared patients who were on anticoagulation (initiated on IPAC or continued outpatient anticoagulation) to patients who were not on anticoagulation. Risk stratification included age, gender, the Elixhauser comorbidity score, vital signs, and D-dimer value [33].

### 2.4 Demographic, clinical, and laboratory variables

The following information was obtained from EHRs: (i) demographic information (age, sex, race/ethnicity); (ii) clinical characteristics in the first 24 h following admission including respiratory distress (defined as < 10 respirations/minute or > 29 respirations/minute), maximum heart rate, minimum systolic blood pressure (SBP-min), minimum oxygen saturation (SpO_2_-min), maximum temperature; (iii) medical history of relevant comorbidities with disease-specific ICD-10 codes as described by our group in other papers [30,31]; (iv) the Elixhauser comorbidity score was calculated for every single patient to represent comorbidity burden [33]; (v) D-dimer values; (vi) patients were also categorized according to having versus not having any of the following cardiovascular, immunological or hematological comorbidities: coronary artery disease, heart failure, prior myocardial infarction (MI), heart valve replacement, cardiac pacemaker, automatic implantable cardioverter device, left ventricular assist device, pulmonary arterial hypertension, atrial fibrillation, supraventricular tachycardia, cerebrovascular disease, lupus anticoagulant, VTE, DIC, heparin-induced thrombocytopenia (HIT), and other hypercoagulable states.

### 2.5 Statistical analysis

All analyses were performed using Stata version 16 or R version 3.6.3. Descriptive data are presented as means ± standard deviations (SD) for the full sample and as means ± standard errors (SE) for all between-group comparisons. Since we did not use the continuous variables as an outcome measure, we did not address normality. Categorical variables are presented as % (*n*). Kaplan-Meier survival curves for time to admission or death were plotted among patients using versus not using anticoagulant therapy as detailed in Fig. 1. Hazard ratios (HR) and 95% confidence intervals (CI) were calculated using multivariable Cox proportional hazards models. The least absolute shrinkage and selection operator (LASSO) model was utilized to select variables from the univariable analysis to be used in multivariable analysis [34]. Follow-up time began at COVID-19 diagnosis and accrued until: (i) date of admission or up to day 45 post-diagnosis, for the admission outcome; or (ii) date of death, or August 27th, 2020 for the death-outcome. In addition, for models predicting hospitalization, we performed a sensitivity analysis by fitting cause-specific competing risk models to account for the competing risk of death [35]. Multivariable models were adjusted for age, sex, self-identified race/ethnicity (as a proxy for social, not biological risk factors), Elixhauser comorbidity score, and the presence/absence of any cardiovascular, immunological or hematological comorbidities. The same analytical approach was utilized among all inpatients for the primary outcome of time to death. We additionally performed a sensitivity analysis among only inpatients with D-dimer values to better assess the potential for confounding by indication.

### 2.6 Role of the funding source

No funding was obtained for this study. Dr. Michael Usher and Dr. Christopher J Tignanelli accessed the data. The decision to submit for publication was a collaborative decision among all the authors.

## 3 Results

Among the 6195 adults included in this analysis, 598 were immediately hospitalized upon diagnosis and 5597 were initially treated as outpatients. The overall case-fatality rate among 6195 adults was 2.8%, with 175 deaths occurring (54 out-of-hospital). Three hundred thirty-one patients were subsequently hospitalized after failing outpatient therapy (5.9%) (Fig. 1). The overall inpatient case-fatality rate was 13% (121 deaths).

The 5597 COVID-19 patients initially treated as outpatients had a mean age of 51 ± 22 years, 57% were women, and 45% self-identified as White, 17 as Black, 9 as Asian, and 11% as others. Among those 5597 patients, patients who failed outpatient management (*n =* 331) and needed to be admitted were ten years older on average, more likely to be male, Hispanic, to have a higher Elixhauser comorbidity score, and to be taking prescription medications reflecting their higher risk profile (Table 1). Results were similar for death (Supplemental Table 1). Supplemental Table 2 summarizes univariable predictors of hospital admission or death. After multivariable adjustment, male gender, non-White race, increased Elixhauser scores, and any cardiovascular, immunological or hematological comorbidities were all associated with increased risk of hospital admission. Age, increased Elixhauser scores, and any cardiovascular, immunological, and hematological comorbidities were associated with an increased risk of death. The HR for hospital admission or death (95% CI) for having any (versus none) of these cardiovascular, immunological, and hematological comorbidities was 2.58 (1.93,3.43), *p* < 0.0001. Additional multivariable predictors of admission or death from competing risk analyses are shown in Table 2.

**Table 1:** **C**haracteristics of 5597 outpatients of M health fairview system from march 4th-August 27th, 2020. Data presented as mean (standard deviation) or % (*n*) in the ‘All’ group. For group comparisons, standard errors are presented parenthetically.

Variable	All	Admitted	Not Admitted	P-value
	% (*n =* 5597)	% (*n =* 331)	% (*n =* 5266)	
Age (years)	50.68 (22.15)	60.50 (1.09)	50.06 (0.31)	< 0.0001
Female	57.1 (3196)	51.4 (170)	57.5 (3026)	0.0341
**Race**				0.0028
White	45.3 (2537)	48.6 (161)	45.1 (2376)	
African American	17.1 (955)	17.5 (58)	17.0 (897)	
Asian	8.7 (489)	12.4 (41)	8.5 (448)	
Hispanic	5.6 (311)	11.2 (37)	5.2 (274)	
Alaskan/Other/Unknown	5.1 (285)	5.4 (18)	5.1 (267)	
**Comorbidities**				
T1DM	2.4 (133)	8.2 (27)	2.0 (106)	< 0.0001
T2DM	15.7 (876)	36.6 (121)	14.3 (755)	< 0.0001
Heart Failure	7.3 (409)	20.2 (67)	6.5 (342)	< 0.0001
Cerebrovascular Disease	6.6 (372)	16.0 (53)	6.1 (319)	< 0.0001
Arrhythmia	12.0 (670)	42.6 (141)	10.0 (529)	< 0.0001
Autoimmune Disease	4.0 (224)	9.4 (31)	3.7 (193)	< 0.0001
Cancer	5.0 (280)	11.8 (39)	4.6 (241)	< 0.0001
COPD	4.5 (250)	12.1 (40)	4.0 (210)	< 0.0001
Hypertension	34.7 (1944)	67.7 (224)	32.7 (1720)	< 0.0001
CKD	10.1 (568)	29.6 (98)	8.9 (470)	< 0.0001
Acute MI	2.3 (129)	9.1 (30)	1.9 (99)	< 0.0001
Cardiovascular, immunological and hematological comorbidities	21.0 (1177)	63.4 (210)	18.4 (967)	< 0.0001
Elixhauser Comorbidity score	2.11 (3.02)	6.27 (0.22)	1.84 (0.04)	< 0.0001
**Medications**				
ACE	3.9 (217)	5.1 (17)	3.8 (200)	0.2818
ARB	3.4 (189)	6.6 (22)	3.2 (167)	0.0012
Any antiplatelet	9.1 (510)	18.4 (61)	8.5 (449)	< 0.0001
Any anticoagulant	2.9 (160)	8.8 (29)	2.5 (131)	< 0.0001
Enoxaparin	0.2 (11)	0.3 (1)	0.2 (10)	1
DOAC	1.5 (82)	4.2 (14)	1.3 (68)	< 0.0001
Warfarin	1.2 (67)	4.2 (14)	1.0 (53)	< 0.0001
Statin	10.8 (603)	21.8 (72)	10.1 (531)	< 0.0001
Metformin	2.5 (142)	4.2 (14)	2.4 (128)	0.0660
Antivirals	0.8 (45)	0.9 (3)	0.8 (42)	1
Nutritional Supplements	12.5 (699)	22.7 (75)	11.8 (624)	< 0.0001

**Table 2:** Multivariable adjusted predictors of hospital admission or death among 5597 outpatients of M health fairview system.

Variable	Admission (*n =* 331)	*P*-value	Death (*n =* 54)	*P*-value
	Hazard Ratio (95% CI)		Hazard Ratio (95% CI)	
Age (1-year increase)	1.00 (0.99, 1.00)	0.5798	1.05 (1.04, 1.06)	< 0.0001
Female	0.79 (0.63, 0.98)	0.0343	0.69 (0.48, 1.00)	0.0493
Race				< 0.0001
White	Reference		Reference	
Other/Unknown	1.75 (1.37, 2.24)	<0.0001	1.10 (0.69, 1.75)	
Comorbidities				
Elixhauser Comorbidity	1.20 (1.17, 1.24)	<0.0001	1.15 (1.10, 1.20)	< 0.0001
score (1 unit increase)				
Cardiovascular,	2.89 (2.04, 4.10)	<0.0001	2.98 (1.75, 5.06)	< 0.0001
immunological and				
hematological				
comorbidities				
Medication				
Outpatient anticoagulant use	0.57 (0.38, 0.87)	0.0086	0.88 (0.50, 1.52)	0.6372

### 3.1 Outpatient anticoagulation therapy and risk of hospital admission or death

**Fig. 2:**
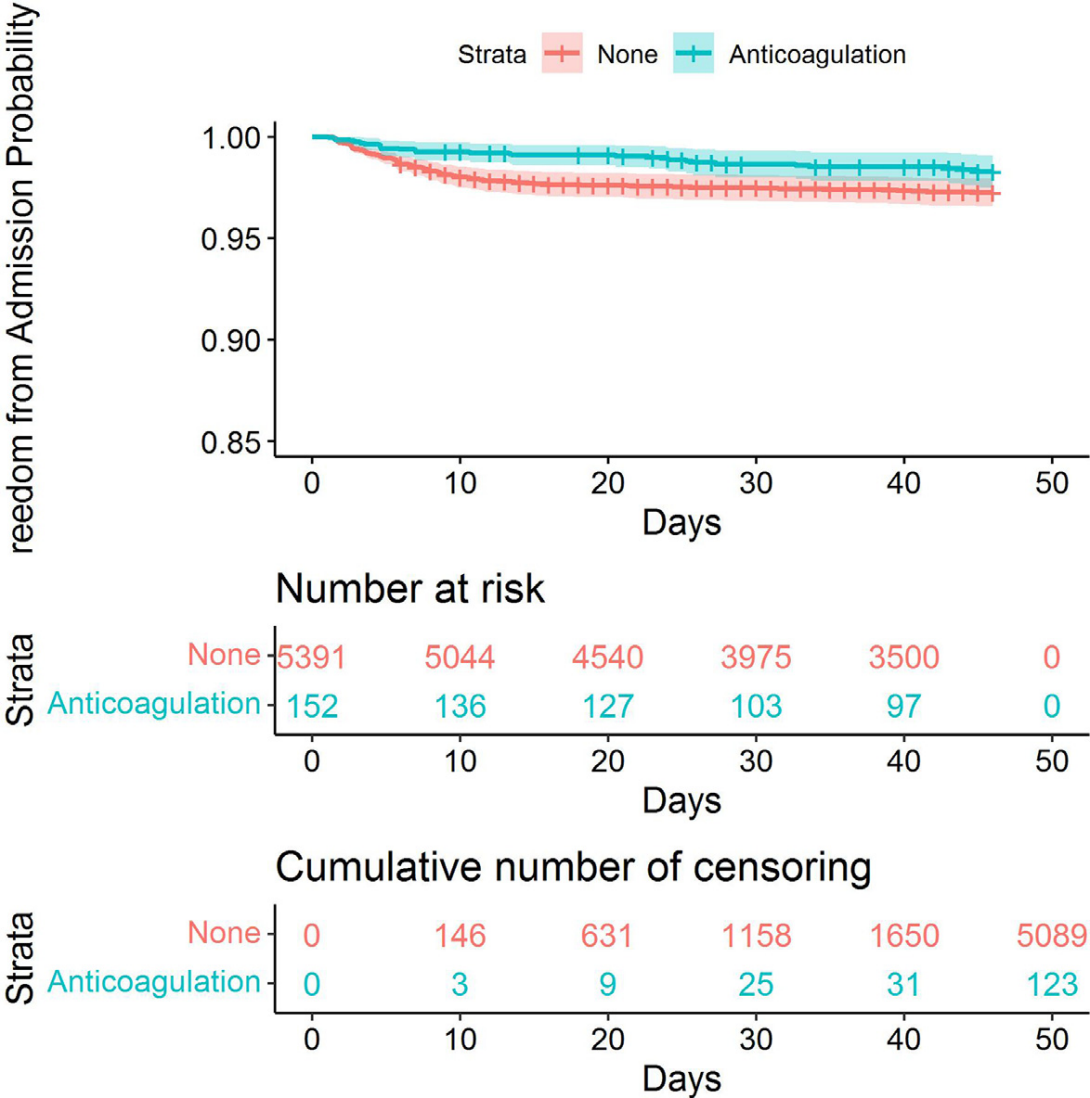
Kaplan-Meier curve describing the probability of admission among COVID-19 outpatients using (blue line) vs. not using (red) anticoagulant therapy.

Among those 5597 patients, 160 patients were on OPAC (2.9%) which consisted of 11 enoxaparin users, 82 DOAC users, and 67 warfarin users. Table 3 summarizes participants’ characteristics among anticoagulant users versus non-users, with anticoagulant users being significantly sicker patients with increased age, higher incidence of cardiovascular, immunological, and hematological comorbidities, and Elixhauser scores. In a multivariable analysis, any OPAC use was associated with a 43% reduction in risk for hospital admission, HR (95% CI) = 0.57 (0.38, 0.86), *p =* 0.007 (Fig. 2). In a multivariable competing risk analysis (considering death as a competing risk), results for OPAC predicting hospital admission were unchanged, HR (95% CI) = 0.57 (0.38, 0.87), *p =* 0.009. After multivariable adjustment, any OPAC use was not empirically associated with mortality, though precision was poor and not statistically significant, HR (95% CI) = 0.88 (0.50, 1.52), *p =* 0.64. Results were unchanged when adjusting for the more concise cardiovascular, immunological, and hematological comorbidities. When considering warfarin, versus DOAC, versus enoxaparin use, all patterns were consistent with the aggregate findings for any anticoagulant use, though precision was poor due to a low number of users for any specific medication class (data not shown).

**Table 3:** Characteristics of 5597 outpatients of M health fairview system according to anticoagulant therapy status data presented as mean (standard deviation) or % (*n*) in the ‘All’ group. For group comparisons, standard errors are presented parenthetically.

Variable	Taking any Anticoagulant	No Anticoagulant	*P*-value
	%(*n =* 160)	%(*n =* 5437)	
Age (years)	70.34 (1.33)	50.10 (0.30)	< 0.0001
Female	51.3 (82)	57.3 (3114)	0.1509
**Race**			< 0.0001
White	75.6 (121)	44.4 (2416)	
African American	8.1 (13)	17.3 (942)	
Asian	3.8 (6)	8.9 (483)	
Hispanic	3.8 (6)	5.6 (305)	
Alaskan/Other/Unknown	1.9 (3)	5.2 (282)	
**Comorbidities**			
T1DM	10.0 (16)	2.2 (117)	< 0.0001
T2DM	41.3 (66)	14.9 (810)	< 0.0001
Heart Failure	52.5 (84)	6.0 (325)	< 0.0001
Cerebrovascular Disease	34.4 (55)	5.8 (317)	< 0.0001
Arrhythmia	75.0 (120)	10.1 (550)	< 0.0001
Autoimmune Disease	17.5 (28)	3.6 (196)	< 0.0001
Cancer	25.0 (40)	4.4 (240)	< 0.0001
COPD	23.1 (37)	3.9 (213)	< 0.0001
Hypertension	84.4 (135)	33.3 (1809)	< 0.0001
CKD	41.9 (67)	9.2 (501)	< 0.0001
Acute MI	16.9 (27)	1.9 (102)	< 0.0001
Cardiovascular, immunological, and hematological comorbidities	93.8 (150)	18.9 (1027)	< 0.0001
Elixhauser Comorbidity	8.26 (0.34)	1.92 (0.04)	< 0.0001
score			
**Medications**			
Warfarin	41.9 (67)	NA	NA
DOAC	51.3 (82)	NA	NA
Enoxaparin	6.9 (11)	NA	NA
Any antiplatelet	28.8 (46)	8.5 (464)	< 0.0001
ACE	13.8 (22)	3.6 (195)	< 0.0001
ARB	15.6 (25)	3.0 (164)	< 0.0001
Statin	60.0 (96)	9.3 (507)	< 0.0001
Metformin	6.9 (11)	2.4 (131)	0.0010
Antivirals	3.8 (6)	0.7 (39)	0.0002
Nutritional Supplements	42.5 (68)	11.6 (631)	< 0.0001

### 3.2 Inpatient anticoagulation therapy and risk of death

There were 929 admitted patients, with 598 patients admitted upon COVID-19 diagnosis and 331 admitted after failing outpatient treatment. Forty-two percent were admitted to the ICU and 20% were placed on mechanical ventilation (48% of those in the ICU). The mortality rates among those with hospital admission, ICU admission, or those treated with a ventilator were 13%, 22%, and 30%, respectively. Among admitted patients, factors associated with death are detailed in Supplemental Table 3.

In hospitalized patients, after multivariable adjustment for age, sex, self-identified race/ethnicity, presence of cardiovascular, immunological, and hematological comorbidities, Elixhauser comorbidity score, maximum temperature, SBP-min, respiratory distress, SpO2-min, patients who did not use any anticoagulation experienced increased mortality, HR (95% CI)= 2.26 (1.17, 4.37), *p =* 0.015 as compared to anticoagulation users. Additional predictors for death are shown in Table 4. The prediction was not enhanced when considering whether anticoagulation was prophylactic, escalated prophylactic, or therapeutic: HRs (95% CIs) for prophylactic/escalated initiation or therapeutic initiation (vs. continuation) was 1.24 (0.73, 2.11) and 1.45 (0.70, 3.02), respectively (both *p*-values > 0.30).

**Table 4:** Predictors of mortality among all inpatients and among the subgroup of patients with D-dimer among 1485 patients of M health fairview system.

	All Inpatients (*n =* 929)		Inpatients with D-dimer (*n =* 556)	
Variable	Death (*n =* 121)	*p*-value	Death (*n =* 73)	*p*-value
	Hazard Ratio (95% CI)		Hazard Ratio (95% CI)	
Age (years)	1.05 (1.03, 1.06)	< 0.0001	1.05 (1.03, 1.07)	< 0.0001
Female	0.75 (0.51, 1.09)	0.1319	0.91 (0.55, 1.51)	0.7214
**Race** White				
Other/Unknown	Reference		Reference	
**Comorbidities** Comorbidity score Any Anticoagulation Indication	0.68 (0.45, 1.03)	0.0678	0.55 (0.32, 0.95)	0.0314
**Anticoagulation changes** Continuation Initiation Never **Biomarkers**	1.05 (1.00, 1.11)	0.0623	1.02 (0.95, 1.10)	0.5543
Oxygen saturation Respiratory distress SBP Temperature	1.63 (0.88, 3.02)	0.1198	1.12 (0.54, 2.29)	0.7653
	Reference		Reference	
	1.27 (0.75, 2.14)	0.3763	0.98 (0.50, 1.90)	0.9477
	2.26 (1.17, 4.37)	0.0151	NA*	NA*
	0.99 (0.97, 1.00)	0.1322	0.99 (0.97, 1.02)	0.6098
	2.10 (1.38, 3.21)	0.0006	1.93 (1.13, 3.32)	0.0169
	0.98 (0.97, 0.99)	<0.0001	0.99 (0.98, 1.00)	0.0103
	1.02 (0.89, 1.15)	0.8163	1.06 (0.90, 1.25)	0.4947
D-dimer	NA	NA	1.06 (1.02, 1.10)	0.0038

*D-dimer tests were not consistently ordered until the change in institutional policy to initiate anticoagulation among all admitted patients. Therefore, all patients in the subgroup with D-dimer lab values received anticoagulation.

### 3.3 D-dimer level and risk of death in hospitalized COVID-19 patients

Among inpatients (*n =* 929), 556 patients had D-dimer levels available. D-dimer levels were not significantly different across all patients on anticoagulation. Those who continued anticoagulation versus patients on prophylactic/escalated anticoagulation versus patients on therapeutic anticoagulation were similar (data expressed as mean (SE)): 1.68(0.38), 2.16(0.17), and 2.76(0.58), respectively (*p =* 0.31). In this subgroup, after adjusting for age, sex, self-identified race/ethnicity, presence of cardiovascular, immunological, and hematological comorbidities, Elixhauser comorbidity score, maximum temperature, SBP-min, respiratory distress, SpO2-min, every 1 mg/dL increase in the D-dimer level was associated with a 6% greater risk of death (HR (95% CI) = 1.06 (1.02,1.10)). In this same multivariable analysis, the HR (95% CI) for death associated with the patients who were initiated on anticoagulation (versus patients who were continued on anticoagulation) was 1.13 (0.58, 2.19), *p =* 0.72. Additional adjustment for D-Dimer levels attenuated this association: HR (95% CI) = 0.98 (0.50,1.90), *p =* 0.95. We were unable to compare patients who were never on anticoagulation to patients who were continued on anticoagulation as there were very few inpatients with D-dimer levels not treated with anticoagulation. We examined the outcomes of patients on anticoagulation versus antiplatelets versus no anticoagulation nor antiplatelets. There was no meaningful difference in outcomes between the antiplatelet group and no anticoagulation or antiplatelets (data not shown).

## 4 Discussion

In this study, individuals using anticoagulation therapy who developed COVID-19 had a 43% lower risk of hospital admission. This study is the largest to date, to our knowledge, to examine all types-anticoagulation in COVID-19 patients in a large and robust dataset. Our study confirms on a larger scale the study by Chocron et al. that showed improved outcomes and reduced admission to intensive care unit in patients on anticoagulation before COVID-19 related hospitalization that was published [36]. It is more generalizable than the study by Rossi et al. given a broader age range in non-cardiac patients [18]. In spite of the significantly worse risk profile of patients on anticoagulation than patients not on anticoagulation in our study, these patients were admitted less often, which may be secondary to the biological protective effect of anticoagulation in COVID-19. This finding confirms our hypothesis that in patients deemed stable enough for outpatient management, anticoagulation can provide a more favorable outcome.

Earlier during the pandemic, before May 2020, there was no widespread consensus about the role of anticoagulation in COVID-19 [[20], [21], [22]]. As such, we had a comparison group of hospitalized COVID-19 patients not on anticoagulation. In this study, any form of anticoagulation offered a favorable mortality outcome in hospitalized COVID-19 patients compared to patients not on any anticoagulation. Our findings were independent of known risk factors for admission and mortality, including age, sex, race/ethnicity, cardiovascular, immunological, and hematological comorbidities, and Elixhauser comorbidity score. Our findings were regardless of the anticoagulation strategy: continued outpatient anticoagulation, or the initiation of prophylactic, escalated prophylactic, or therapeutic anticoagulation during hospitalization. This study also confirms data from Paranjpe et al. and Tang et al. for potentially favorable outcomes for hospitalized COVID-19 patients on anticoagulation in a larger sample size [20,21]. It also confirms the data of Nadkarni et al. and Billett et al. that showed no difference in outcomes between different anticoagulation strategies [22,27]. There have been concerns about the potential for adverse bleeding events negating the benefits of anticoagulation in hospitalized COVID-19 patients. However, serious bleeding events tend to be relatively uncommon in this population based on prior data [22,27]. While our study was underpowered to study bleeding complications, the comparable mortality rates between those initiated on anticoagulation or were continued on anticoagulation are reassuring. Similar to other studies, those with elevated D-dimer levels had an increased risk of death [29,37].

At the time of this publication, multiple randomized trials are examining the use of anticoagulation in COVID-19. A recently published open-label clinical trial randomized 615 hospitalized COVID-19 patients with elevated D-dimer into receiving therapeutic anticoagulation while hospitalized followed by rivaroxaban for 30 days versus prophylactic anticoagulation while hospitalized only. There was increased bleeding among patients randomized to therapeutic anticoagulation with no improvement in outcomes [38]. In another clinical trial, 600 COVID-19 intensive care unit patients were randomized to escalated prophylactic anticoagulation versus prophylactic anticoagulation. There was no difference in clinical outcomes between the two groups [39]. Even more recently, two new articles published results of randomized controlled trials for anticoagulation in COVID-19 patients. One article based on data of 2219 non critically ill COVID-19 patients showed improved outcomes with therapeutic anticoagulation compared to prophylactic or escalated dose anticoagulation [40]. The caveat was that 20% of the patients in the intervention arm did not receive therapeutic anticoagulation versus 26% of the patients in the control arm received escalated prophylactic anticoagulation. The second article was based on data of 1089 critically ill COVID-19 patients, did not show a difference in outcomes between patients randomized to therapeutic anticoagulation versus the control arm [41]. Again, the caveat was that 22% of patients in the intervention arm did not receive therapeutic anticoagulation and 51% of patients in the control arm received escalated prophylactic anticoagulation. Results are awaited for other trials examining anticoagulant use among COVID-19 outpatients. In due course, these trials will provide rigorous evidence regarding the benefits of anticoagulant therapy in the context of COVID-19 and insights regarding the optimal anticoagulant type, dosage, and duration of therapy.

Unfortunately, many patients with an indication for anticoagulation are not receiving therapy. In a study of almost 100,000 veterans with nonvalvular atrial fibrillation, only 49% were on anticoagulation even after the introduction of DOAC [42]. Similar results were observed among a cohort of Medicare patients [43]. Even if patients were prescribed anticoagulation, suboptimal adherence to anticoagulation use exposes patients to a higher risk of thrombotic events, as noted in a study of more than half a million atrial fibrillation patients [44]. Our study demonstrates that, in addition to reducing the risk for thrombotic complications of atrial fibrillation, OPAC reduces the risk for hospital admission in those diagnosed with COVID-19. This finding can help improve adherence of patients eligible for anticoagulation to their medications as a public health approach to minimize COVID-19 hospitalization as we, unfortunately, approach the next pandemic surge.

Inflammatory cytokines and the subsequent endothelium inflammation observed in COVID-19 likely contribute to the observed risk for thrombosis. Several plausible pathways have been proposed. The genes most significantly upregulated in the lungs of COVID-19 patients are SERPINS E1, F1, G1 that encode for antiplasmin, tissue plasminogen activator inhibitor 1, and C1-esterase inhibitor, as well as the prothrombinase FGL2 (fibrinogen like protein 2). All of these possess significant prothrombotic activity. The relationship between inflammation and hypercoagulability is well established in the literature [45]. In addition, coagulation factors and platelets have been shown to modulate the host immune response to widespread infections, but how this may lead to an increased risk for thrombosis is unknown [46]. Several mechanisms have been proposed to explain how anticoagulation therapy can improve COVID-19 outcomes. Anticoagulants may lead to a reduction of COVID-19 associated macrovascular thrombosis, including a reduction of pulmonary embolism, thrombotic stroke, and hemodialysis circuit clots [47]. Alternatively, anticoagulants may reduce microvascular thrombosis, including alveolar and glomerular capillary microthrombi [48,49].

Limitations of this study include the relatively low number of patients on anticoagulation (*n =* 160, which is 2.9%) despite a large number of enrolled patients (*n =* 5597) which may have led to an imbalance of statistical power. However, this percentage of patients on anticoagulation is consistent if not slightly higher than expected in the USA [50]. Although the crude HR for the protective effect of anticoagulation was 0.57 for hospitalizations in outpatients using anticoagulation, there is the possibility of unmeasured confounding. For instance, patients receiving anticoagulants could be more health-conscious with an increased likelihood of taking other health-promoting measures that might have helped minimize the severity of the infection and improved their outcomes. For example, these patients may have maintained more strict social distancing when infected thus had less viral load that led to their disease. Higher viral load has been linked to worse COVID-19 outcomes [51]. Our study also had an incomplete adjustment for prognosis at baseline with a lack of body mass index and hemoglobin A1C levels among diabetics. Both markers are beyond the categorical data that the Elixhauser comorbidity score is based upon. Both characteristics have been linked to the severity of outcomes in COVID-19 patients [52,53]. Another point to consider is the possibility that anticoagulation treatment is so powerful at reducing poor outcomes, it overcomes the poor prognosis of patients on those medications. From that perspective, it is conceptually possible to see a crude HR < 1.0.

Furthermore, the exact indications and dosage for anticoagulation were inferred from diagnostic codes and were not fully reported, which may have contributed to confounding. Additionally, among inpatients, the reasons for withholding or initiating anticoagulation may not have been well captured in our multivariable model and could be an essential source of residual confounding. Our data was extracted from a single healthcare system, and we cannot rule out the possibility that outpatients were hospitalized outside of our healthcare system. However, it is unlikely that hospitalization outside of our system would include a large number of patients, which would bias results. Mortality data were informed by state-level death certificates, which would identify deaths outside of our system within our state, so again if patients were admitted to another healthcare system and died, those would have been captured. Despite our study's large size, we were underpowered to detect potentially subtle protective effects of specific types of anticoagulation for the mortality outcome given the relatively low number of patients who passed away. We were also underpowered to look at thrombosis and adverse bleeding outcomes. Although aspirin has been linked to improved outcomes of hospitalized COVID-19 patients, we did not examine the impact of aspirin specifically in this study [54]. As such results from large randomized clinical trials, which randomize both known and unknown confounders, are eagerly awaited to define dose, type, and duration of anticoagulation for COVID-19 patients in outpatient, inpatient, and post-hospital discharge settings as well as the role of aspirin.

An essential strength of our study is the large sample size, powered to detect modest associations between anticoagulation and hospitalization. Our large healthcare system includes patients across a broad range of localities, from urban to rural, and is likely to be highly generalizable to many health systems. This study also represents real-world data showing beneficial anticoagulation effects in older and sicker populations who are on outpatient anticoagulation.

In conclusion, we have found a reduced risk for hospitalization among patients using outpatient anticoagulation for at least 90 days before a diagnosis of COVID-19. We also observed a modest but not statistically significant trend towards reduced mortality in outpatient COVID-19 patients. To date, there is no consensus on the type of anticoagulant, dosage, or duration of therapy. Randomized controlled trials for anticoagulation therapy among both inpatients and outpatients are urgently awaited to address these critical questions for COVID-19 patients.

## Funding

None

## Data sharing statement

Data will be made available upon request to the authors.

## Declaration of Competing Interest

Dr. Tignanelli has a relationship (contract/grant) with the Gates Foundation and Minnesota Partnerships to conduct randomized controlled trial(s) of Losartan in COVID-19, outside the submitted work. Dr. Haslbauer and Dr. Tzankov received funding support from the Botnar Research Centre for Child Health Foundation Research Grant on COVID-19 for all their COVID-19 related research, outside the submitted research. Dr. Lutsey received NIH grants outside the submitted work. Dr. Shah received a MHealth Fairview Learning health system K12 grant, travel award for HTRS colloquium and ASH Medical educator institute award, and has a ASH system-based hematology committee leadership role. All theother authors have nothing to disclose

## Appendix Supplementary materials

Image, application 1
