# Supplementary material for: Outpatient and inpatient anticoagulation therapy and the risk for hospital admission and death among COVID-19 patients
Source: eClinicalMedicine. 2021 Sep 24;41:101139. doi: 10.1016/j.eclinm.2021.101139 (PMC8461367; doi:10.1016/j.eclinm.2021.101139)
Supplement: Supplementary file 1 [file mmc1.docx]

**Supplemental Data**

**Number of elements:** 3 supplemental tables

**Titles of elements:**

1. **Supplemental Table 1.** Characteristics of Outpatients by Survival Status of M Health System from March 4th – August 27th, 2020.
2. **Supplemental Table 2.** Supplemental Table 2. Univariable Predictors of Hospital Admissions and Death among Outpatients of M Health System from March 4th – August 27th, 2020
3. **Supplemental Table 3.** Univariable Predictors of Death among 1,485 inpatients of M Health System

**Supplemental Table 1. Characteristics of Outpatients by Survival Status of M Health System from March 4th – August 27th, 2020**

Data presented as mean (standard deviation) or %(n) in the ‘All’ group. For group comparisons, standard errors are presented parenthetically.

| **Variable** | **Died**  **% (n=116)** | **Survived**  **% (n=5481)** | **P-value** |
| --- | --- | --- | --- |
| Age (years) | 78.35 (1.48) | 50.09 (0.30) | <0.0001 |
| Female | 50.9 (59) | 57.2 (3133) | 0.2015 |
| **Race** |  |  | 0.0024 |
| White | 68.1 (79) | 44.8 (2458) |  |
| African American | 7.8 (9) | 17.3 (946) |  |
| Asian | 6.9 (8) | 8.8 (481) |  |
| Hispanic | 4.3 (5) | 5.6 (306) |  |
| Alaskan/Other/Unknown | 6.0 (7) | 5.1 (278) |  |
| **Comorbidities**  T1DM | 9.5 (11) | 2.2 (122) | <0.0001 |
| T2DM | 43.1 (50) | 15.1 (826) | <0.0001 |
| Heart Failure | 37.9 (44) | 6.7 (365) | <0.0001 |
| Cerebrovascular Disease | 29.3 (34) | 6.2 (338) | <0.0001 |
| Arrhythmia | 48.3 (56) | 11.2 (614) | <0.0001 |
| Autoimmune Disease | 12.1 (14) | 3.8 (210) | <0.0001 |
| Cancer | 12.9 (15) | 4.8 (265) | 0.0002 |
| Comorbidity score | 7.17 (0.35) | 2.00 (0.04) | <0.0001 |
| COPD | 16.4 (19) | 4.2 (231) | <0.0001 |
| Hypertension | 81.0 (94) | 33.8 (1850) | <0.0001 |
| CKD | 46.6 (54) | 9.4 (514) | <0.0001 |

| Acute MI | 8.6 (10) | 2.2 (119) | <0.0001 |
| --- | --- | --- | --- |
| Cardiovascular, immunological and hematological comorbidities | 79.3 (92) | 19.8 (1085) | <0.0001 |
| **Medications** |  |  |  |
| Any Antiplatelet | 29.3 (34) | 8.7 (476) | <0.0001 |
| Any Anticoagulant | 14.7 (17) | 2.6 (143) | <0.0001 |
| Enoxaparin | 0.9 (1) | 0.2 (10) | 0.5644 |
| DOAC | 9.5 (11) | 1.3 (71) | <0.0001 |
| Coumadin | 4.3 (5) | 1.1 (62) | 0.0073 |
| ACE | 6.9 (8) | 3.8 (209) | 0.1445 |
| ARB | 7.8 (9) | 3.3 (180) | 0.0173 |
| Statin | 31.0 (36) | 10.3 (567) | <0.0001 |
| Metformin | 1.7 (2) | 2.6 (140) | 0.7915 |
| Antivirals | 0.0 (0) | 0.8 (45) | 0.6494 |
| Nutritional Supplements | 21.6 (25) | 12.3 (674) | 0.0045 |

**Supplemental Table 2. Univariable Predictors of Hospital Admissions and Death among Outpatients of M Health System from March 4th – August 27th, 2020**

Data presented as mean (standard deviation) or %(n) in the ‘All’ group. For group comparisons, standard errors are presented parenthetically.

| **Variable** | **Admission**  **(n=)** | **P-value** | **Death**  **(n=)** | **P-value** |
| --- | --- | --- | --- | --- |
|  | **HR (95%CI)** |  | **HR (95%CI)** |  |
| Age (1-year increase) | 1.02 (1.02, 1.02) | <0.0001 | 1.06 (1.05, 1.07) | <0.0001 |
| Female | 0.78 (0.63, 0.97) | 0.0273 | 0.77 (0.53, 1.11) | 0.1554 |
| **Race**  White | Reference |  | Reference |  |
| Other/Unknown | 1.16 (0.92, 1.46) | 0.2052 | 0.46 (0.30, 0.71) | 0.0004 |
| **Comorbidities** |  |  |  |  |
| T1DM | 3.68 (2.49, 5.46) | <0.0001 | 3.50 (1.87, 6.55) | <0.0001 |
| T2DM | 3.21 (2.57, 4.02) | <0.0001 | 3.69 (2.55, 5.34) | <0.0001 |
| Heart Failure | 3.44 (2.63, 4.50) | <0.0001 | 6.80 (4.66, 9.93) | <0.0001 |
| Cerebrovascular Disease | 2.85 (2.13, 3.83) | <0.0001 | 5.32 (3.56, 7.95) | <0.0001 |
| Arrhythmia | 5.98 (4.81, 7.44) | <0.0001 | 6.01 (4.15, 8.69) | <0.0001 |
| Autoimmune Disease | 2.59 (1.79, 3.75) | <0.0001 | 2.98 (1.70, 5.22) | 0.0001 |
| Cancer | 2.63 (1.88, 3.68) | <0.0001 | 2.49 (1.44, 4.29) | 0.0011 |
| Elixhauser Comorbidity | 1.26 (1.23, 1.28) | <0.0001 | 1.27 (1.23, 1.31) | <0.0001 |
| score (1 unit increase) |  |  |  |  |
| COPD | 3.08 (2.21, 4.29) | <0.0001 | 3.65 (2.22, 5.98) | <0.0001 |
| Hypertension | 4.07 (3.23, 5.12) | <0.0001 | 7.19 (4.51, 11.45) | <0.0001 |
| CKD | 3.95 (3.12, 5.01) | <0.0001 | 6.75 (4.67, 9.75) | <0.0001 |
| Acute MI | 4.52 (3.10, 6.57) | <0.0001 | 3.33 (1.73, 6.43) | 0.0003 |

| Cardiovascular, immunological and hematological comorbidities  **Medications** | 7.18 (5.74, 8.99) | <0.0001 | 13.06 (8.31, 20.51) | <0.0001 |
| --- | --- | --- | --- | --- |
| Any anticoagulant | 3.52 (2.41, 5.15) | <0.0001 | 6.41 (3.73,11.01) | <0.0001 |
| Any antiplatelet | 2.36 (1.78, 3.11) | <0.0001 | 4.36 (2.89, 6.57) | <0.0001 |
| ACE | 1.36 (0.84, 2.22) | 0.2155 | 1.84 (0.89, 3.76) | 0.0976 |
| ARB | 2.13 (1.38, 3.28) | 0.0006 | 2.31 (1.17, 4.57) | 0.0158 |
| None  Warfarin | Reference  4.13 (2.42, 7.06) | <0.0001 | Reference  4.35 (1.71, 11.05) | 0.0020 |
| DOAC | 3.32 (1.94, 5.68) | <0.0001 | 8.35 (4.29, 16.25) | <0.0001 |
| Enoxaparin | 1.58 (0.22,11.23) | 0.6493 | 5.39 (0.68, 42.55) | 0.1099 |
| Statin | 2.38 (1.83, 3.09) | <0.0001 | 3.56 (2.40, 5.29) | <0.0001 |
| Metformin | 1.71 (1.00, 2.92) | 0.0494 | 0.62 (0.15, 2.52) | 0.5046 |
| Antivirals | 1.12 (0.36, 3.49) | 0.8470 | NA | NA |
| Nutritional Supplements | 2.11 (1.63, 2.73) | <0.0001 | 1.81 (1.16, 2.82) | 0.0092 |

**Supplemental Table 3. Univariable Predictors of Death among 1,485 inpatients of M Health System**

|  | **Death**  **(n=121)** | **P-value** |
| --- | --- | --- |
| **Variable** | **HR (95%CI)** |  |
| Age (years) | 1.05 (1.04, 1.06) | <0.0001 |
| Female | 0.69 (0.48, 0.99) | 0.0450 |
| BMI  **Race** | 0.98 (0.95, 1.00) | 0.0545 |
| White | Reference |  |
| Other/Unknown | 0.52 (0.35, 0.76) | 0.0008 |
| **Comorbidities** |  |  |
| T1DM | 1.56 (0.93, 2.60) | 0.0905 |
| T2DM | 1.54 (1.08, 2.20) | 0.0172 |
| Heart Failure | 2.32 (1.60, 3.36) | <0.0001 |
| Cerebrovascular Disease | 2.77 (1.91, 4.01) | <0.0001 |
| Arrhythmia | 2.07 (1.44, 2.99) | <0.0001 |
| Autoimmune Disease | 1.69 (1.01, 2.82) | 0.0447 |
| Cancer | 1.24 (0.74, 2.11) | 0.4158 |
| Comorbidity score | 1.15 (1.10, 1.20) | <0.0001 |
| COPD | 2.35 (1.57, 3.51) | <0.0001 |
| Hypertension | 3.22 (1.90, 5.46) | <0.0001 |
| CKD | 2.92 (2.04, 4.17) | <0.0001 |
| Acute MI | 1.94 (1.21, 3.10) | 0.0059 |

| Any anticoagulation | 5.11 (2.93, 8.92) | <0.0001 |
| --- | --- | --- |
| indication  **Outpatient Medications** |  |  |
| **Anticoagulation** |  |  |
| Continuation | Reference |  |
| Initiation | 0.46 (0.28, 0.74) | 0.0017 |
| Never | 0.45 (0.24, 0.82) | 0.0099 |
| ACE | 0.79 (0.35, 1.80) | 0.5787 |
| ARB | 1.69 (1.01, 2.83) | 0.0439 |
| Statin | 2.51 (1.76, 3.60) | <0.0001 |
| Metformin | 1.09 (0.51, 2.34) | 0.8233 |
| Antivirals | 0.71 (0.10, 5.12) | 0.7382 |
| Nutritional Supplements | 1.16 (0.77, 1.76) | 0.4731 |
| **Biomarkers** |  |  |
| CA | 0.73 (0.66, 0.81) | <0.0001 |
| CO2 | 1.03 (1.01, 1.06) | 0.0110 |
| Creatinine | 1.12 (1.04, 1.21) | 0.0029 |
| HCT | 0.94 (0.91, 0.97) | 0.0004 |
| Heart Rate | 1.01 (1.00, 1.02) | 0.0178 |
| Neutrophil/lymphocyte | 1.02 (1.01, 1.03) | 0.0005 |
| ratio |  |  |
| Oxygen saturation | 0.97 (0.96, 0.98) | <0.0001 |
| RDW | 1.17 (1.09, 1.26) | <0.0001 |

| Respiratory distress | 3.12 (2.12, 4.58) | <0.0001 |
| --- | --- | --- |
| SBP | 0.98 (0.97, 0.98) | <0.0001 |
| Temperature | 1.04 (0.93, 1.16) | 0.5302 |
| WBC | 1.01 (1.00, 1.01) | 0.1327 |

**Caption for Supplementary Material:**

1. **Supplemental Table 1**. Characteristics of Outpatients by Survival Status of M Health System from March 4th – August 27th, 2020.Data presented as mean (standard deviation) or %(n) in the ‘All’ group. For group comparisons, standard errors are presented parenthetically.
2. **Supplemental Table 2**. Supplemental Table 2. Univariable Predictors of Hospital Admissions and Death among Outpatients of M Health System from March 4th – August 27th, 2020. Data presented as mean (standard deviation) or %(n) in the ‘All’ group. For group comparisons, standard errors are presented parenthetically.
3. **Supplemental Table 3.** Univariable Predictors of Death among 1,485 inpatients of M Health System
